# The Association Between Polycystic Ovary Syndrome and Metabolic Syndrome in Adolescents: a Systematic Review and Meta-analysis

**DOI:** 10.1007/s43032-022-00864-8

**Published:** 2022-02-02

**Authors:** Leyi Fu, Ningning Xie, Fan Qu, Jue Zhou, Fangfang Wang

**Affiliations:** 1 Women’s Hospital, School of Medicine, Zhejiang University, 1 Xueshi Road, Hangzhou, 310006 China; 2 College of Food Science and Biotechnology, Zhejiang Gongshang University, Hangzhou, 310018 China

**Keywords:** Adolescent, Polycystic ovary syndrome, Metabolic syndrome

## Abstract

The association between polycystic ovary syndrome (PCOS) and metabolic syndrome (MetS) is not widely recognized or properly assessed in adolescents. The aim of this study was to conduct a systematic review and meta-analysis to provide reliable results concerning MetS development in adolescents with PCOS. We searched studies published in PubMed, Medline, and Web of Science from January 2010 to December 2020. The quality of studies was assessed by the Newcastle–Ottawa Scale (NOS), and the data analysis was performed with Stata 14.0. Twelve articles were finally included in the systematic review and meta-analysis. The results suggested that adolescents with PCOS have more than three times the odds of having MetS than controls (OR 3.32, 95% CI [2.14, 5.14]). Obese adolescents with PCOS also had a higher risk of MetS than those with obesity but without PCOS (OR 3.97, 95% CI [1.49, 10.53]). Compared to those without PCOS, systolic blood pressure was higher in adolescents with PCOS (weighted mean difference (WMD) 3.85, 95% CI [1.73, 5.97]), while diastolic blood pressure was higher only in girls with PCOS who had a normal weight (WMD 3.52, 95% CI [1.57, 5.48]). The levels of triglycerides were higher in obese adolescents with PCOS than in those with obesity but without PCOS (WMD 27.84, 95% CI [10.16, 45.51]). PCOS could increase the frequency of MetS by influencing blood pressure and lipid metabolism independent of obesity as early as the adolescent period. Thus, clinicians should perform early interventions in adolescents with PCOS and follow up the relevant indicators of MetS to decrease the risk of poor long-term prognosis.

**Supplementary Information:**

The online version contains supplementary material available at 10.1007/s43032-022-00864-8.

## Introduction

Polycystic ovary syndrome (PCOS) is a common endocrine disorder in women of reproductive age with the prevalence from 5 to 15% and is the primary cause of anovulatory female infertility [1, 2]. The common manifestations of PCOS include ovulation dysfunction, hyperandrogenism, polycystic ovarian morphology, obesity, and insulin resistance [3], which could present beginning in adolescence [4]. Metabolic syndrome (MetS) is a complex syndrome characterized by elevated blood pressure (BP), fasting blood glucose (FBG), and triglycerides (TGs) but reduced high-density lipoprotein (HDL), as well as central obesity [5]. It was found that the prevalence of MetS increased gradually in the past decades, and its clinical manifestation could start as early as adolescence [6].

There seems to be an overlap of pathogenesis between PCOS and MetS as both of these conditions could involve insulin resistance (IR) [7]. Several studies reported that women with PCOS had an increased risk of MetS [8–10]. It was reported that the prevalence of MetS in adolescents with PCOS was likely higher than that in adolescents without PCOS [6, 11], but the effect of obesity was not accounted for properly, and an analysis of the components of MetS also needs to be performed in detail. Therefore, we conducted a systematic review and meta-analysis of studies published in the past decade and related to the association between PCOS and MetS in adolescents, which aimed to provide valid and comprehensive analysis results for clinicians to enhance the awareness of early and holistic management of adolescents with PCOS.

## Methods

### Search Strategy

This systematic review and meta‐analysis was conducted according to the Preferred Reporting Items for Systematic Reviews and Meta-Analyses (PRISMA) statement [12]. We searched studies published in PubMed, Medline, and Web of Science from January 2010 to December 2020 using the keywords ((polycystic ovar* syndrome) OR (polycystic ovar* disease)) AND ((adolescen*) OR (girl)) AND ((metabolic syndrome) OR (syndrome X) OR (insulin* resistan*) OR (obes*) OR (lipid*)). The full review protocol was registered with PROSPERO with the registration number of CRD 42,021,240,836.

### Study Selection

Based on the PRISMA statement [12], two authors independently screened the titles, abstracts, and full-text articles to select studies published in the English language that compared the prevalence of MetS between adolescents with PCOS and non-PCOS controls or included all the components of MetS in adolescents with PCOS and non-PCOS controls. Any disagreements were resolved by discussion with a third review author. Studies including reviews, meta-analyses, letters, comments, animal studies, or clinical case reports were excluded.

### Data Extraction

Two authors independently extracted the data from each eligible study. Data retrieved included the name of the first author, year of publication, type of study design, country of study, diagnostic criteria of PCOS and MetS, sample size for cases and controls, age and body mass index (BMI), the prevalence of MetS in cases and controls, and the components of MetS including BP, waist circumference (WC), TGs, HDL, and FBG.

### Quality Assessment

The quality of the included studies was evaluated by the same two authors using the Newcastle–Ottawa Quality Assessment Scale (NOS). The NOS is used to assess studies in terms of selection bias, comparability bias, and outcome bias [13]. Selection bias could be awarded zero to four stars, and the maximum number of stars for comparability bias and outcome bias is two and three, respectively. Studies were considered good or fair quality if they scored at least two stars for selection bias, one star for comparability bias part, and two stars for outcome bias [8].

### Statistical Analysis

MetS was assessed as a dichotomous variable, and the odds ratio (OR, 95% confidence interval [CI]) was used to present the analysis outcome. In addition, the components of MetS were assessed as continuous variables, which were expressed by weighted mean differences (WMDs, 95% CIs). Heterogeneity between the studies was evaluated by using *I*^2^ tests and *P* values: if *P* > 0.01 and *I*^2^ < 50%, indicating acceptable heterogeneity, the fixed-effect model was used; if *P* ≤ 0.01 and *I*^2^ > 50%, indicating substantial heterogeneity, the random effects model was used [14]. Sensitivity analysis was conducted to explore the influence of individual studies on the overall pooled effect by calculating the remaining pooled effect after excluding one study. In addition, subgroup analysis according to BMI was conducted if significant heterogeneity existed. Publication bias was evaluated by using Begg’s test and Egger’s test. If *P* < 0.05, indicating significant asymmetry, the results might be questionable. Data analyses were conducted using Stata (version 14.0).

## Results

### Search Results

**Fig. 1:**
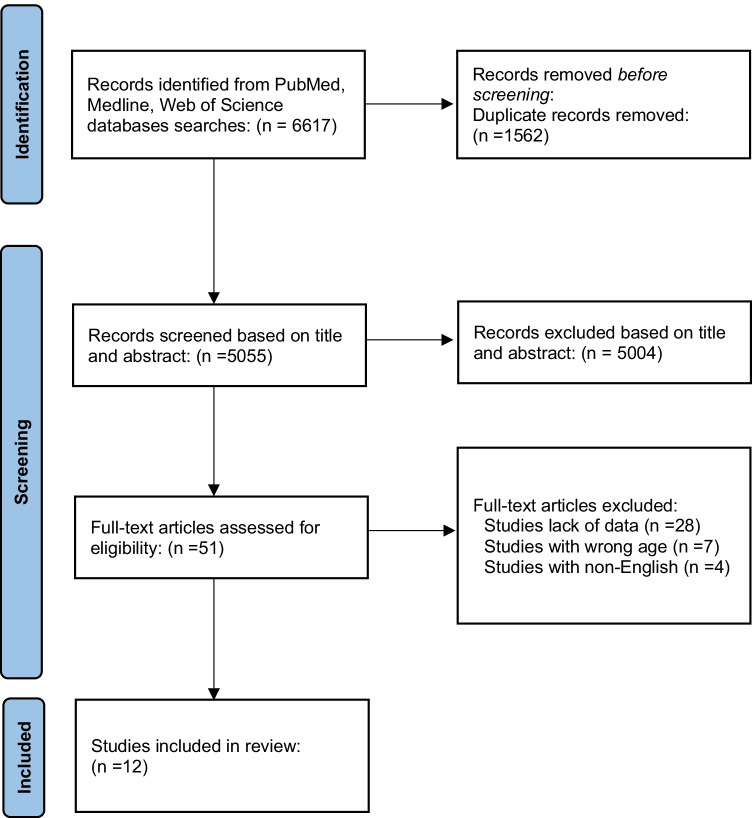
Flow chart of the literature search results

The search strategy yielded 6,617 studies from a total of three databases. Figure 1 shows the flow chart of the literature search and selection process. After removing 1562 duplicates, 5,004 studies were excluded because they did not fulfil the selection criteria according to the title or abstract. After a full-text review of 51 potential studies, we further excluded 28 studies due to lack of essential data (any data of the components of MetS including BP, WC, TGs, HDL, and FBG), 7 studies with participants out of the age range between 10 and 20 years, and 4 studies that were not written in the English language. A total number of 12 studies were included in the systemic review and further meta-analysis.

### Characteristics of Included Studies

Table S1 presents the characteristics of the included studies, and Table S2 presents the quality of the included studies using the NOS. Of 12 articles, ten studies [15–24] compared the prevalence of MetS in adolescents with PCOS and controls, and the other 2 studies [25, 26] compared all the components of MetS in adolescents with PCOS and controls. Eleven articles were case–control studies, and the other one was a prospective cohort study. Regarding the quality of the studies, four studies were of good quality, and the other 8 studies were of fair quality.

For the definition of PCOS, five studies used the definition of the Rotterdam criteria [15, 17, 19, 21, 25], four studies used the definition of the European Society for Human Reproduction and Embryology/American Society for Reproductive Medicine (ESHRE/ASRM) criteria [18, 22, 24, 26], two studies used the definition of the National Institutes of Health (NIH) criteria [20, 23], and the last one used the definition of the Androgen Excess Society (AES) 2006 criteria [16]. Different diagnostic criteria included different phenotypes of PCOS, which lead to different compositions of primary characteristics of PCOS including hyperandrogenaemia, hirsutism, oligoanovulation, and polycystic ovaries [27, 28]. For the definition of MetS, six studies used the definition of the International Diabetes Federation (IDF) criteria [15, 17, 18, 20, 21, 24]. One study used the definition of the National Cholesterol Education Program Adult Treatment Panel (NCEP ATP) III criteria [23]. One study used the ‘2009 joint interim criteria’ [16]. One study used the Weiss criteria [19], and the remaining study used the modified Cook criteria [22].

In addition, Rahmanpour et al. [20], Güven et al. [25] and Han et al. [23] matched BMI by dividing individuals into normal weight and obese groups. Nandalike et al. [19] and Hughan et al. [26] carried out research on obese adolescents; however, Aydin et al. [22] selected normal weight adolescents for research. The rest of the included studies did not match BMI in adolescents.

### Systematic Review and Meta-analysis

#### Prevalence of Metabolic Syndrome in Adolescents

Ten of the included studies showed that the prevalence of MetS in adolescents with PCOS varied from 4.08 to 60.78%. Three studies presented a significantly higher prevalence of MetS in PCOS patients than controls [16, 20, 22]; four studies also presented a moderately higher prevalence of MetS in PCOS patients than controls [17, 19, 21, 24]; the *P* value was not mentioned in the remaining 3 studies [18, 23, 26].

**Fig. 2:**
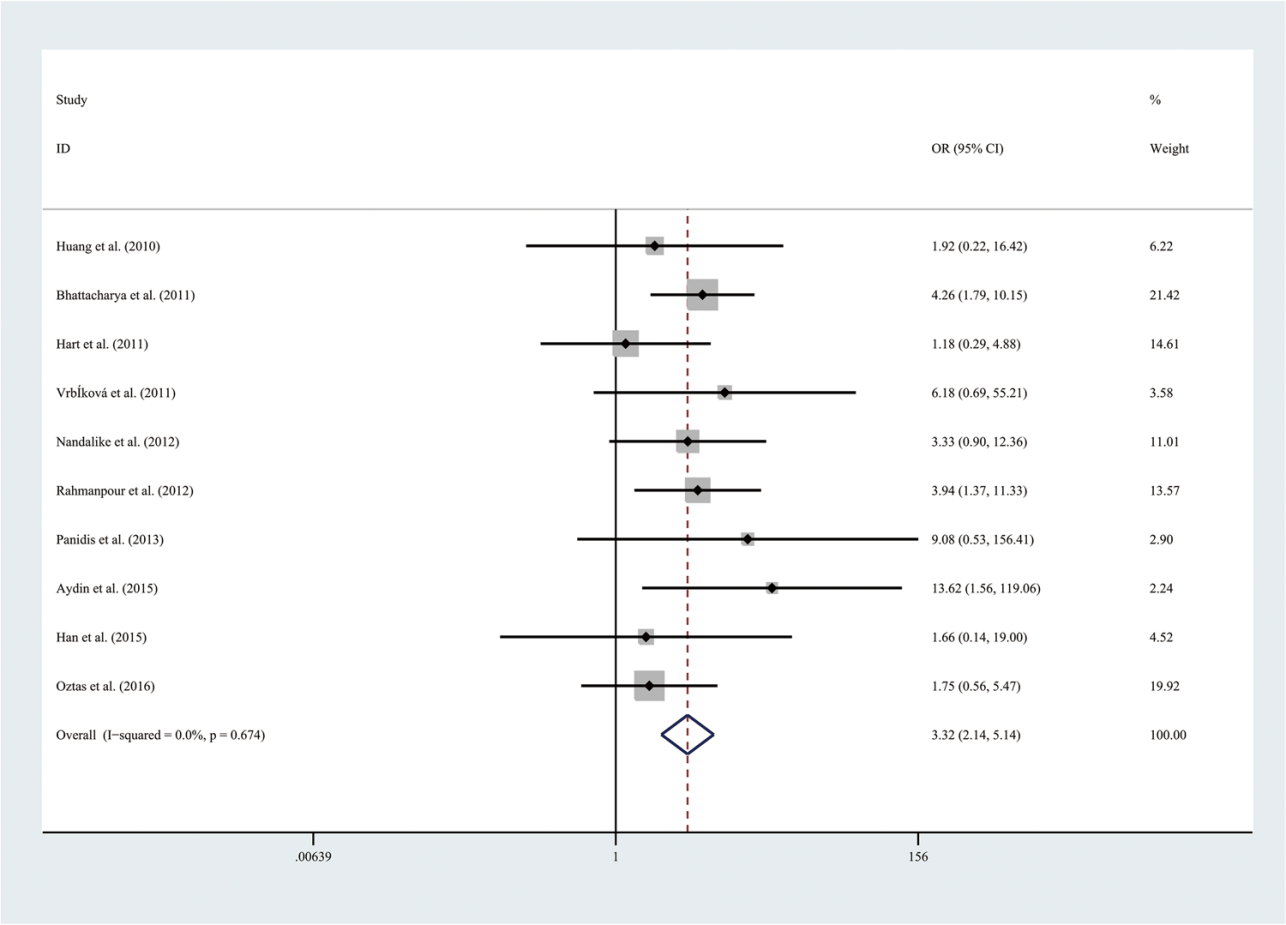
Meta-analysis of MetS prevalence in adolescents with and without PCOS. OR, odds ratio; 95% CI, 95% confidence interval

**Fig. 3:**
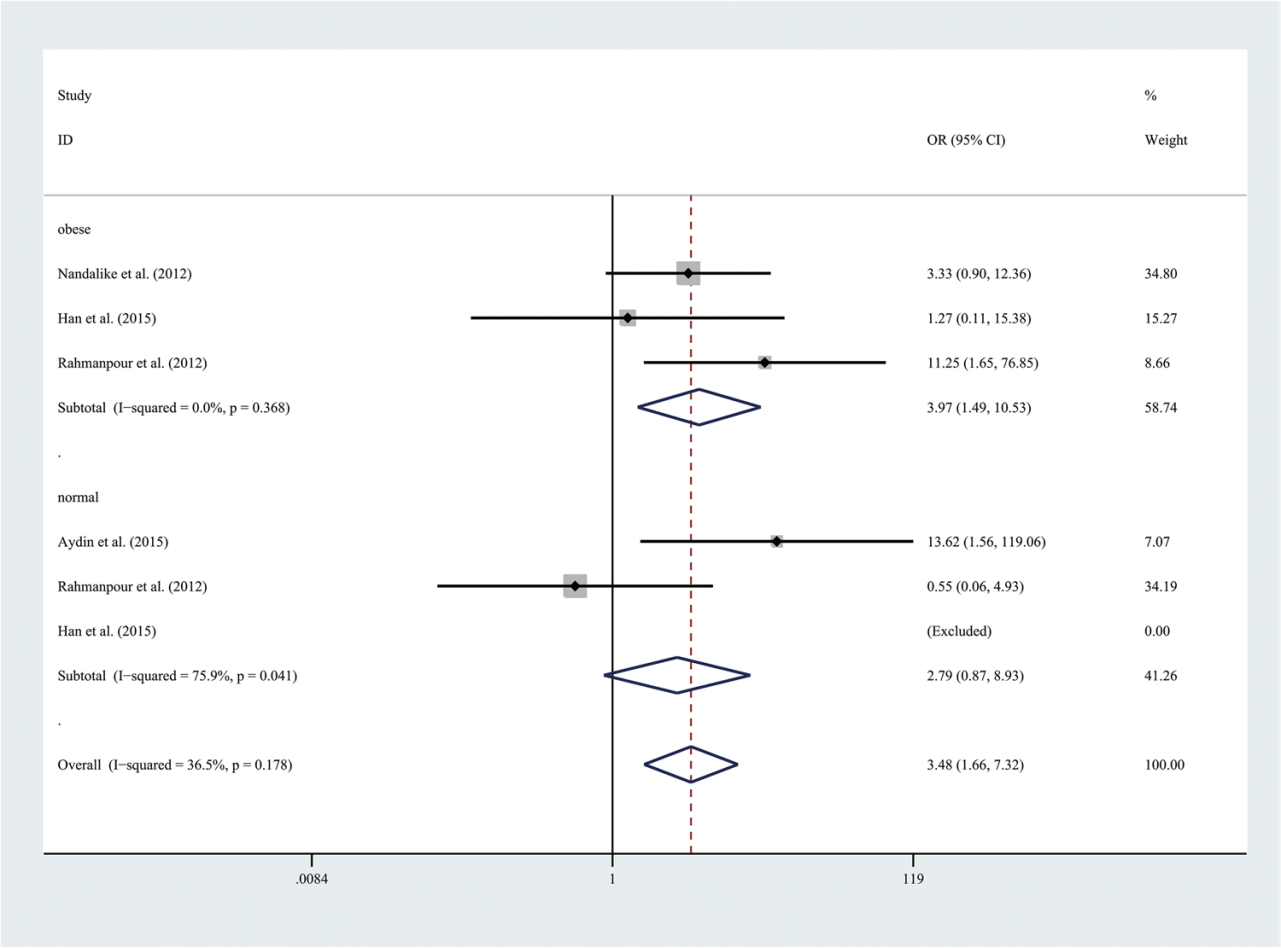
Subgroup analysis with different weight of MetS prevalence in adolescents with and without PCOS. OR, odds ratio; 95% CI, 95% confidence interval. The weight of adolescents included obese and normal; only the studies with different weight were taken to analyse

Therefore, the results of the meta-analysis of 10 studies with a fixed-effect model showed that the risk of MetS was more than three times higher in adolescents with PCOS than in adolescents without PCOS (OR 3.32, 95% CI [2.14, 5.14]), with low statistical heterogeneity (*I*^2^ = 0%, *P* = 0.674) (Fig. 2). To exclude the influence of weight, subgroup analyses with the random effect model were performed in 4 BMI-matched studies (Fig. 3). The above results showed that the prevalence of MetS was higher in obese adolescents with PCOS than in obese controls (OR 3.97, 95% CI [1.49, 10.53]), with low statistical heterogeneity (*I*^2^ = 0%, *P* = 0.368). Nevertheless, the prevalence of MetS was comparable between normal weight adolescents with PCOS and controls.

#### Blood Pressure Level in Adolescents

Nine of the included studies compared the blood pressure level between adolescents with PCOS and controls. One study found that both the systolic and diastolic blood pressures of adolescents with PCOS were higher than those of controls [22], three studies only found higher SBP in adolescents with PCOS [19, 21, 26], and Rahmanpour et al. [20] only found higher DBP in adolescents with PCOS. The remaining 4 studies found no significant statistical differences between groups [16, 18, 23, 25].

**Fig. 4:**
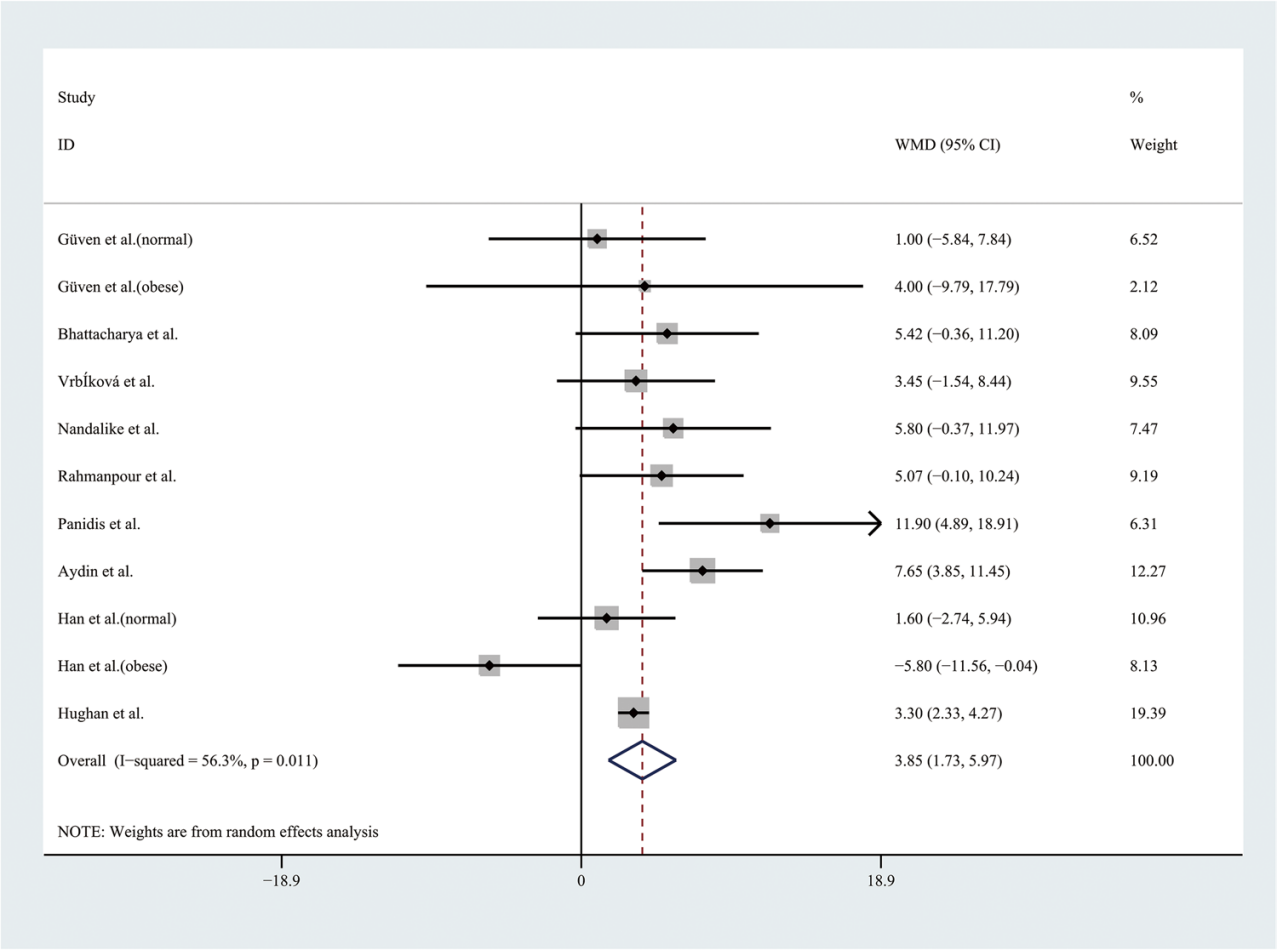
Meta-analysis of SBP level in adolescents with and without PCOS. SBP, systolic blood pressure; WMD, weighted mean difference; 95% CI, 95% confidence interval. Two studies recording the level of SBP for adolescents of different weight were analysed separately

**Fig. 5:**
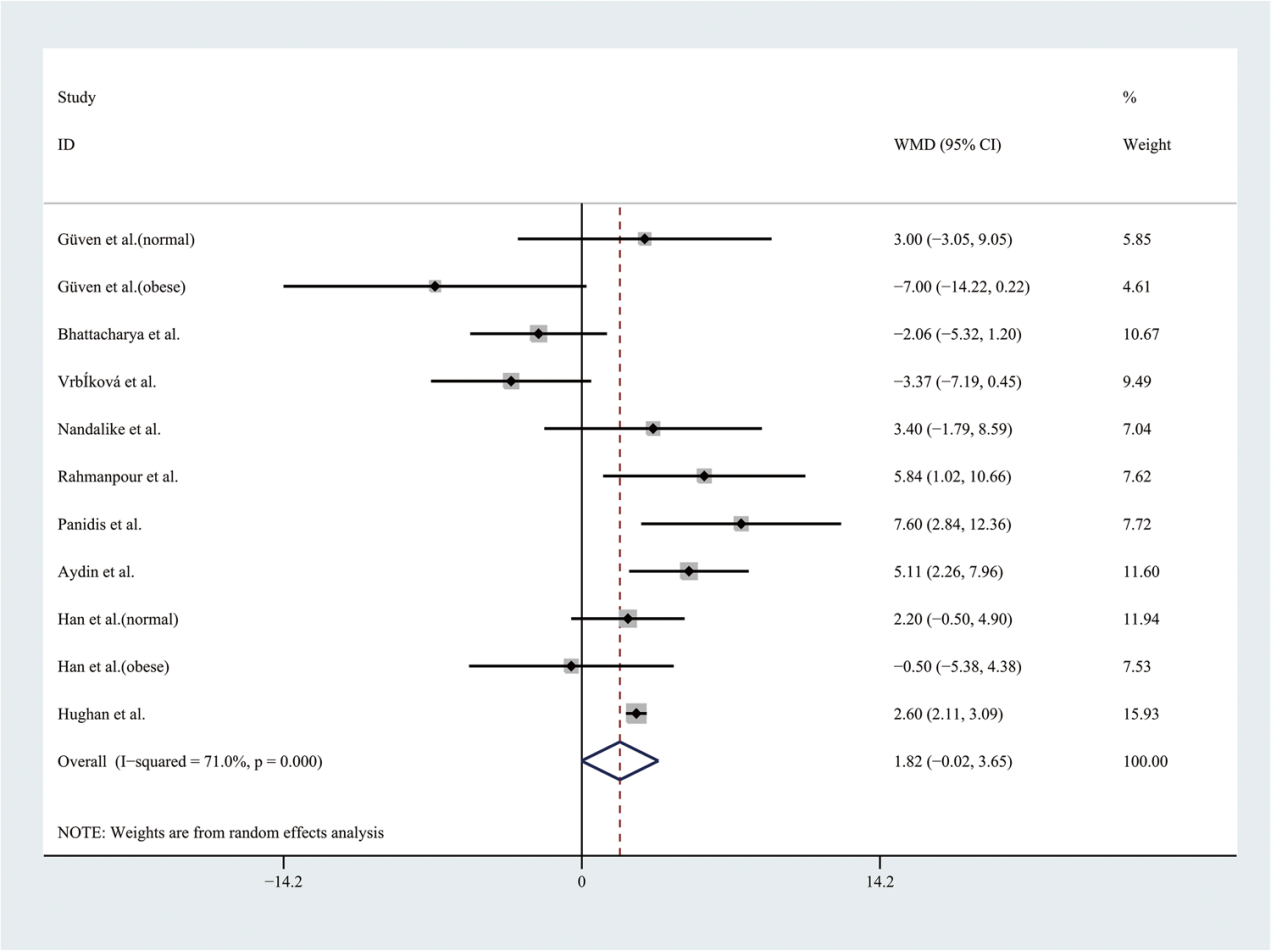
Meta-analysis of DBP level in adolescents with and without PCOS. DBP, diastolic blood pressure; WMD, weighted mean difference; 95% CI, 95% confidence interval. Two studies recording the level of DBP for adolescents of different weight were analysed separately

**Fig. 6:**
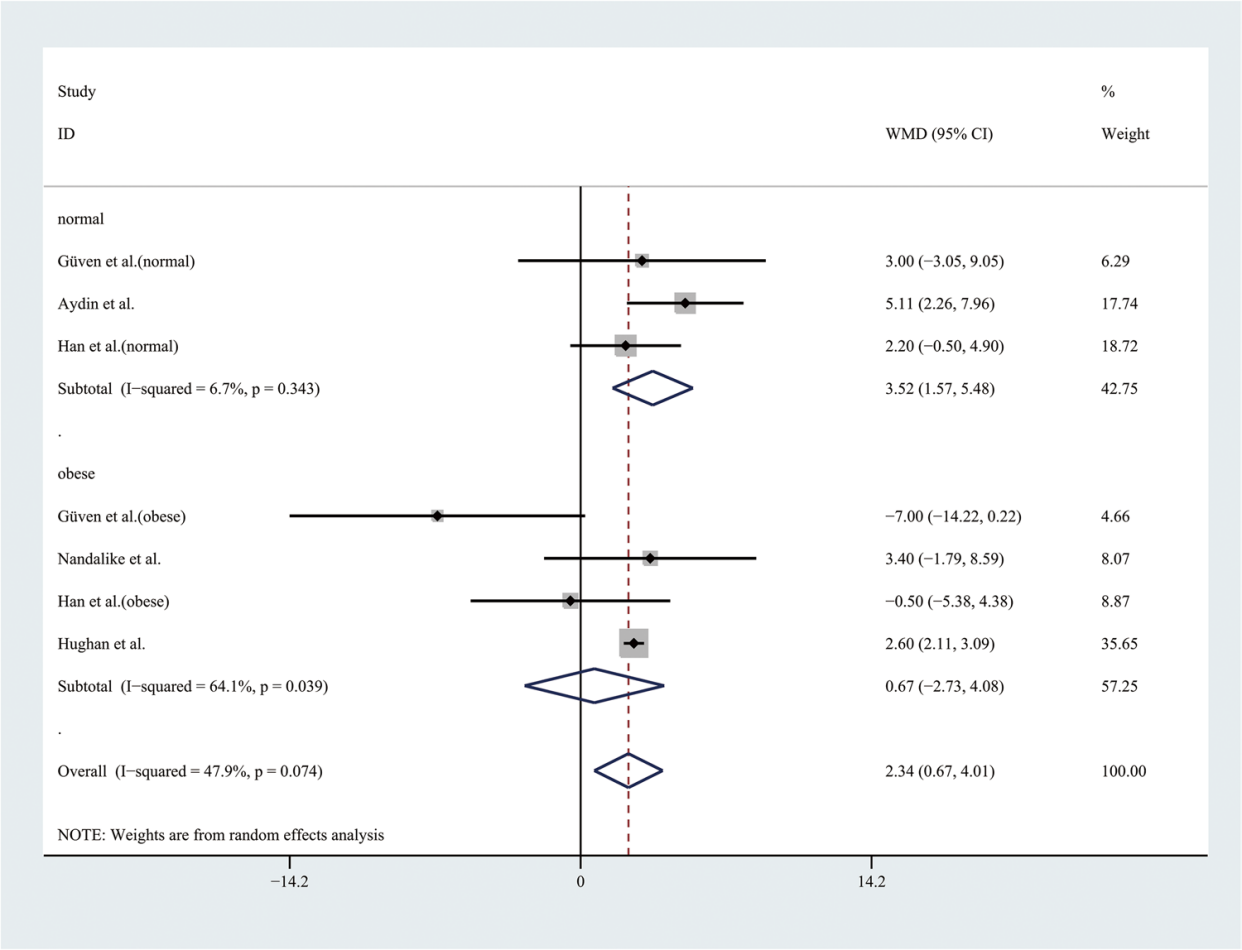
Subgroup analysis with different weight of DBP level in adolescents with and without PCOS. WMD, weighted mean difference; 95% CI, 95% confidence interval. The studies with different weight were taken to analyse

Therefore, the results of the meta-analysis with a random effect model of 9 studies showed that the level of SBP was higher in adolescents with PCOS than in controls (WMD 3.85, 95% CI [1.73, 5.97]), with moderate statistical heterogeneity (*I*^2^ = 56.3%, *P* = 0.011) (Fig. 4). However, the level of DBP was not significantly different between groups, with significant statistical heterogeneity (*I*^2^ = 71%, *P* < 0.001) (Fig. 5). To alleviate the heterogeneity, subgroup analyses with the random effect model were performed with 5 BMI-matched studies (Fig. 6). The results showed that the level of DBP was higher in normal weight adolescents with PCOS than in controls (WMD 3.52, 95% CI [1.57, 5.48]), with low statistical heterogeneity (*I*^2^ = 6.7%, *P* = 0.343). However, there were no significant differences in the obese groups.

#### Central Obesity in Adolescents

Nine of the included studies compared the waist circumference between adolescents with PCOS and controls. Four studies found that adolescents with PCOS had higher WC than controls [16, 20, 21, 26], and Güven et al. [25] found that only obese adolescents with PCOS had higher WC than obese controls. The remaining 4 studies found no significant differences between groups [18, 22–24].

**Fig. 7:**
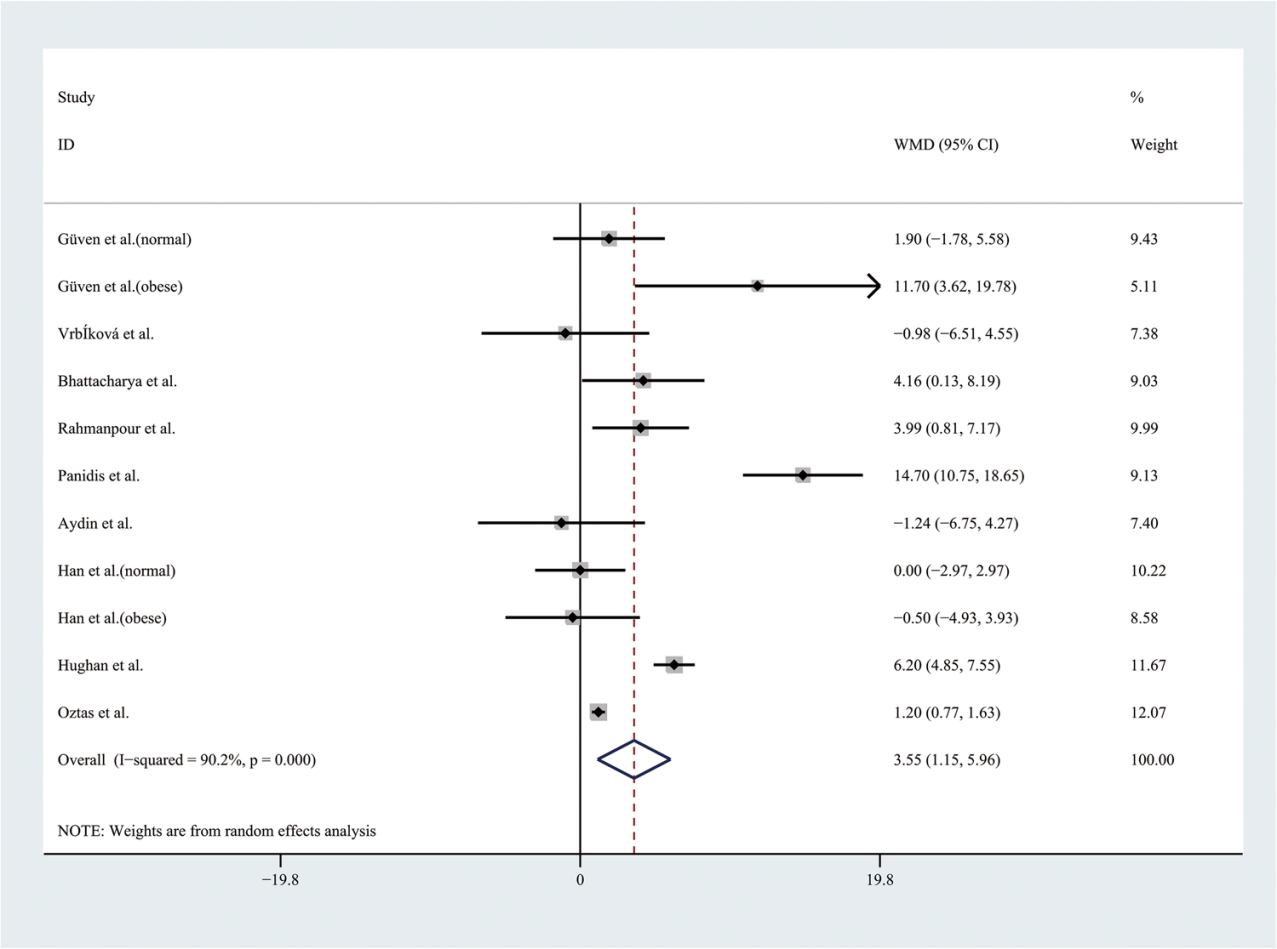
Meta-analysis of WC in adolescents with and without PCOS. WC, waist circumference; WMD, weighted mean difference; 95% CI, 95% confidence interval. Two studies recording the WC for adolescents of different weight were analysed separately

**Fig. 8:**
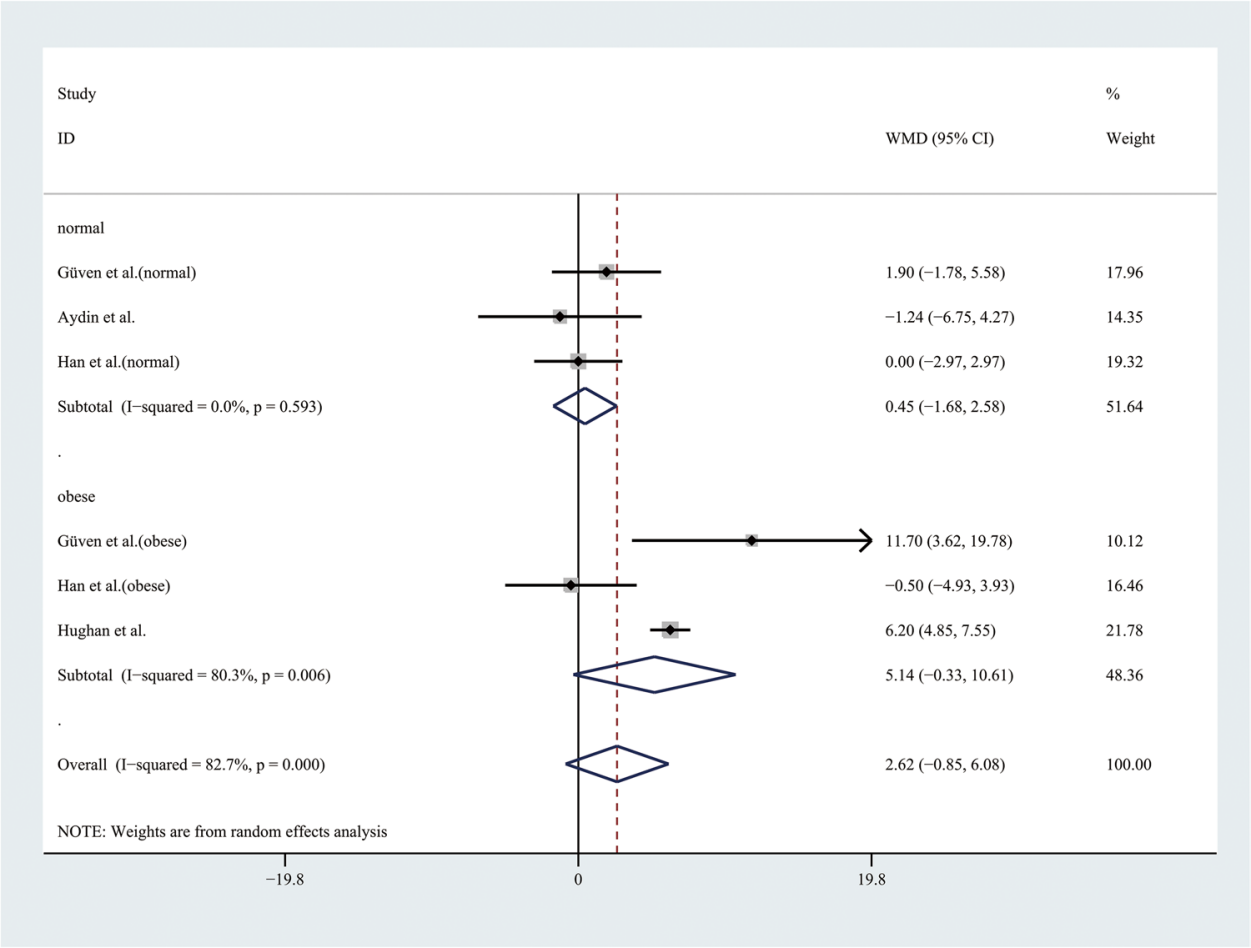
Subgroup analysis with different weight of WC in adolescents with and without PCOS. WC, waist circumference; WMD, weighted mean difference; 95% CI, 95% confidence interval. The studies with different weight were taken to analyse

Therefore, the results of the meta-analysis with a random effect model of 9 studies showed that adolescents with PCOS had higher WC than controls (WMD 3.55, 95% CI [1.15, 5.96]), with significant statistical heterogeneity (*I*^2^ = 90.2%, *P* < 0.001) (Fig. 7). To alleviate the heterogeneity, subgroup analyses with the random effect model were performed in 4 BMI-matched studies (Fig. 8). However, there were no significant differences between the groups.

#### Lipid Level in Adolescents

Ten of the included studies compared the levels of TGs and HDL between adolescents with PCOS and controls. Three studies found that adolescents with PCOS had higher TGs levels than controls [20, 22, 26], but the remaining 7 studies did not find significant difference between groups [16, 18, 19, 21, 23–25]. In addition, 4 studies found that adolescents with PCOS had lower HDL levels than controls [18, 19, 24, 26], and the remaining 6 studies found no significant differences between groups [16, 20–23, 25]. One study [22] was excluded from the meta-analysis due to a lack of availability of standard deviation data.

**Fig. 9:**
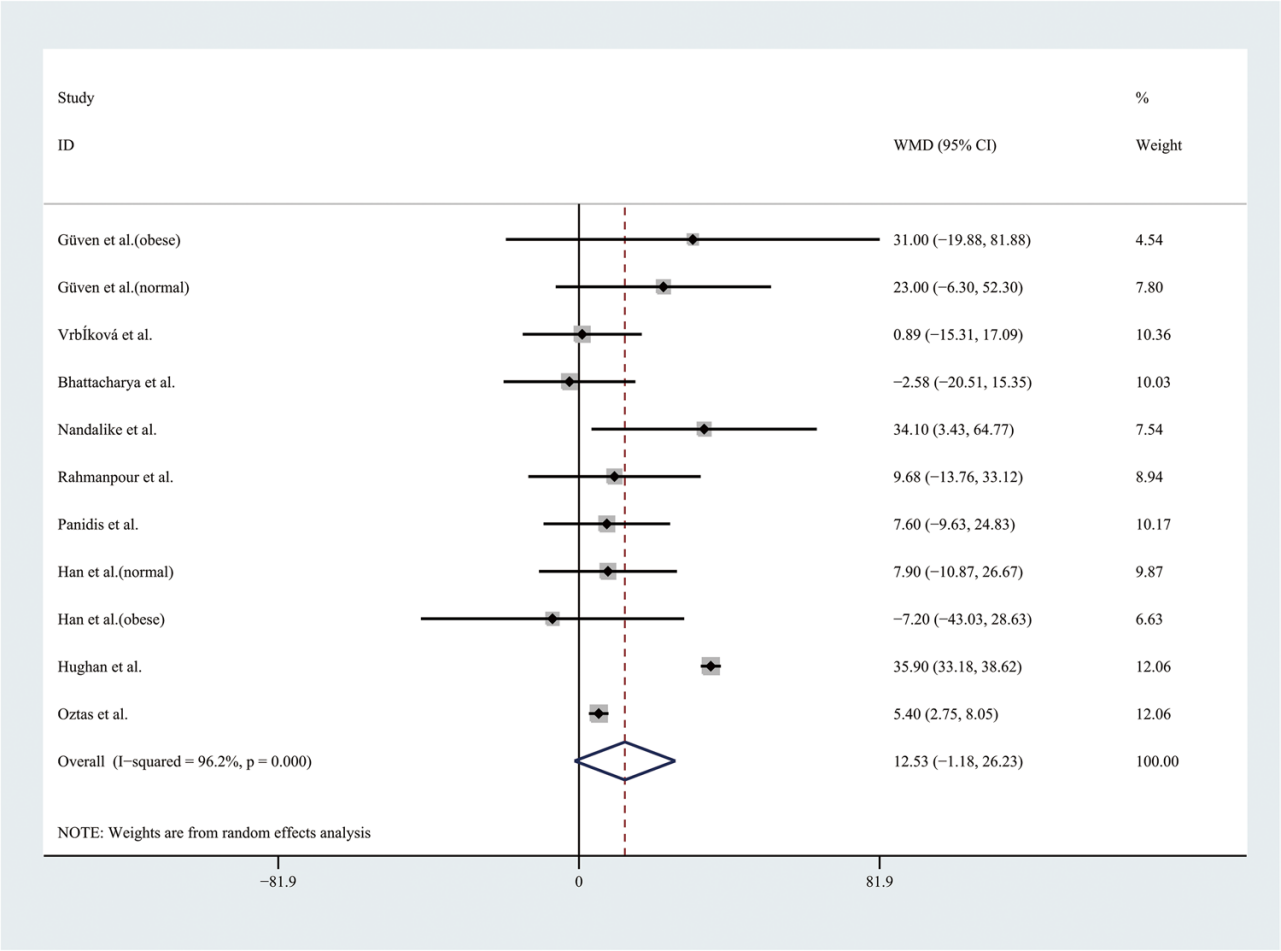
Meta-analysis of TG level in adolescents with and without PCOS. TGs, triglycerides; WMD, weighted mean difference; 95% CI, 95% confidence interval. Two studies recording the TG for adolescents of different weight were analysed separately

**Fig. 10:**
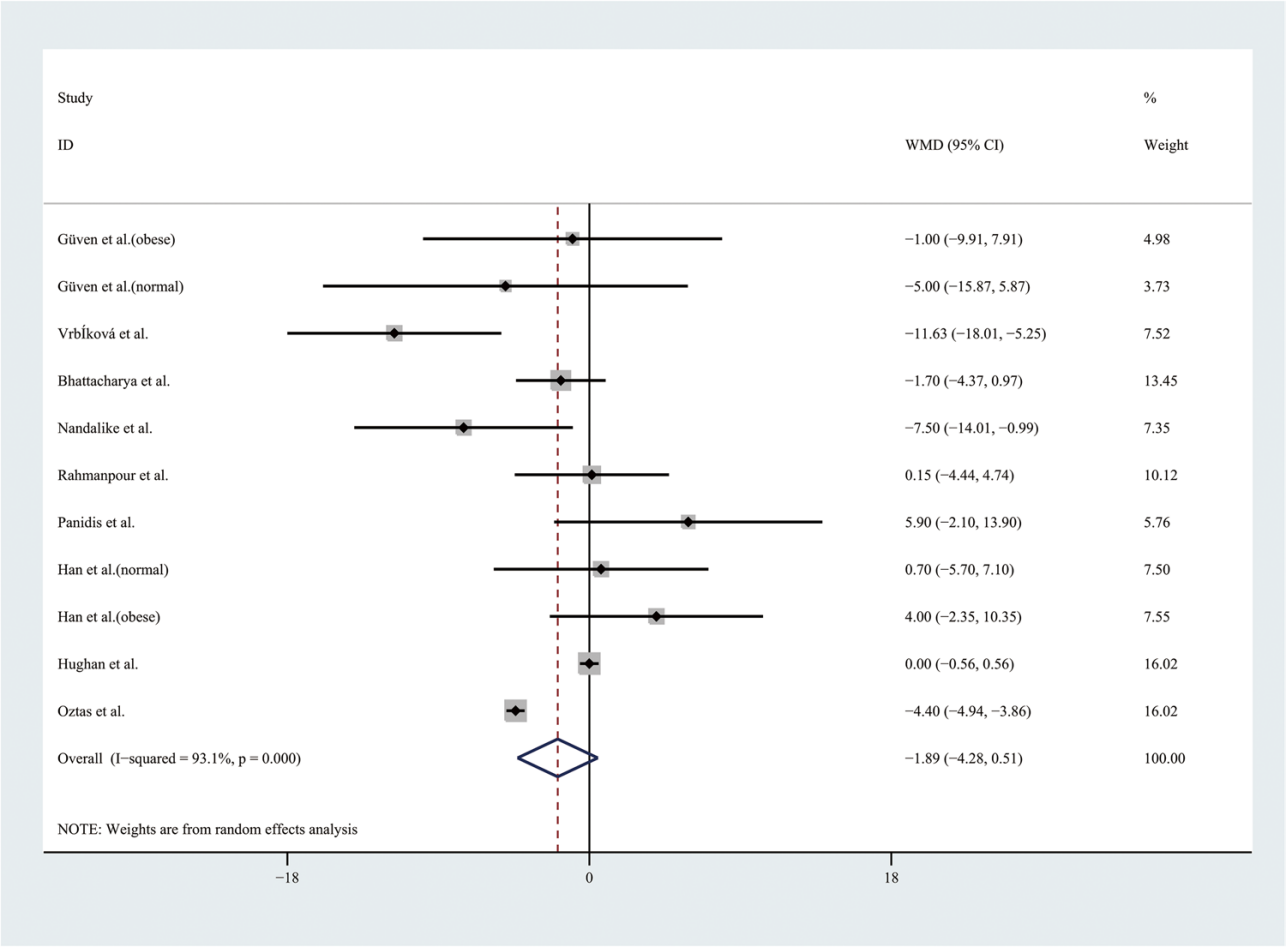
Meta-analysis of HDL level in adolescents with and without PCOS. HDL, high-density lipoprotein; WMD, weighted mean difference; 95% CI, 95% confidence interval. Two studies recording the HDL for adolescents of different weight were analysed separately

**Fig. 11:**
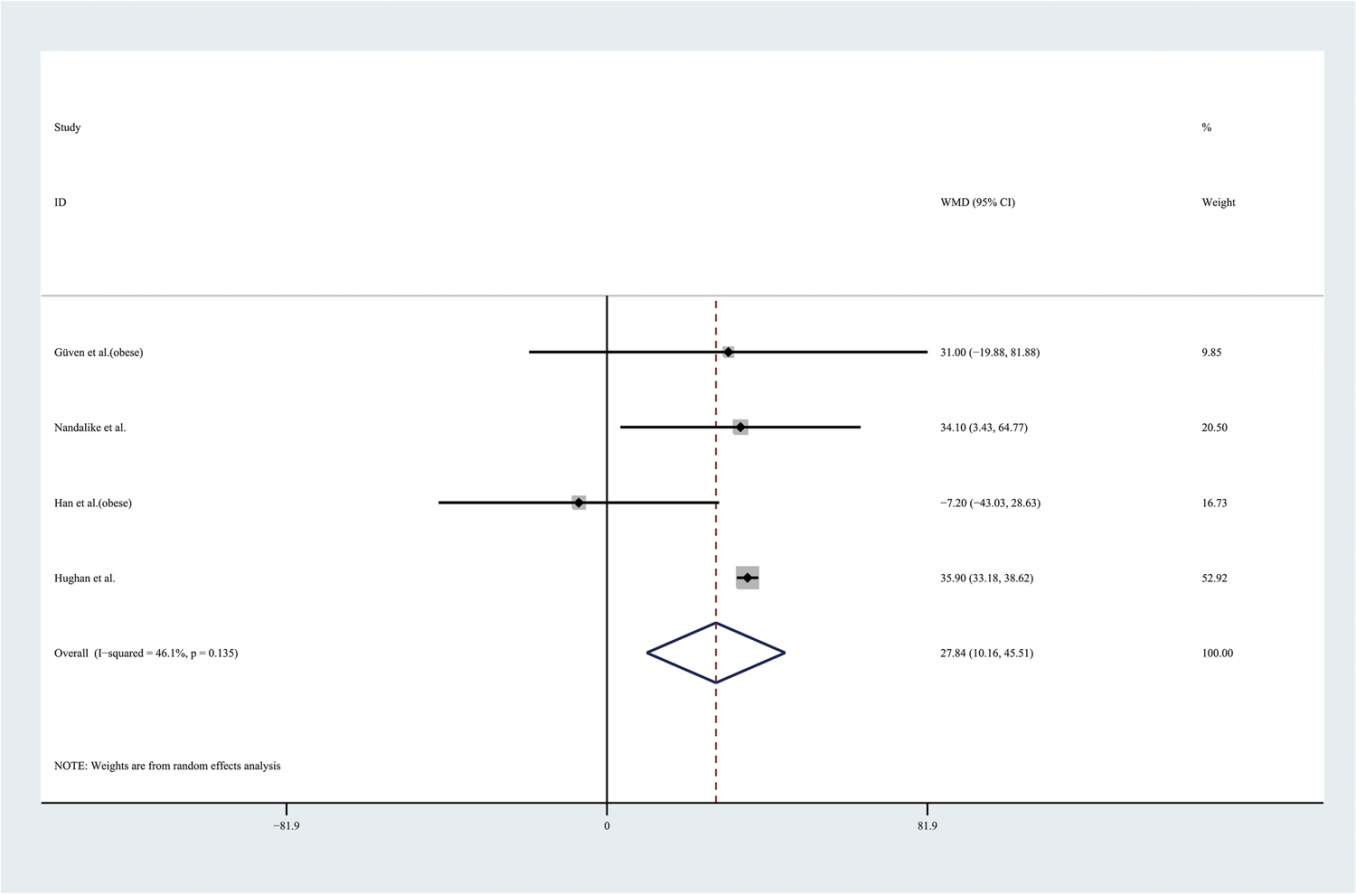
Subgroup analysis of TG level in obese adolescents with and without PCOS. TGs, triglycerides; WMD, weighted mean difference; 95% CI, 95% confidence interval. The studies with obese weight were taken to analyse

**Fig. 12:**
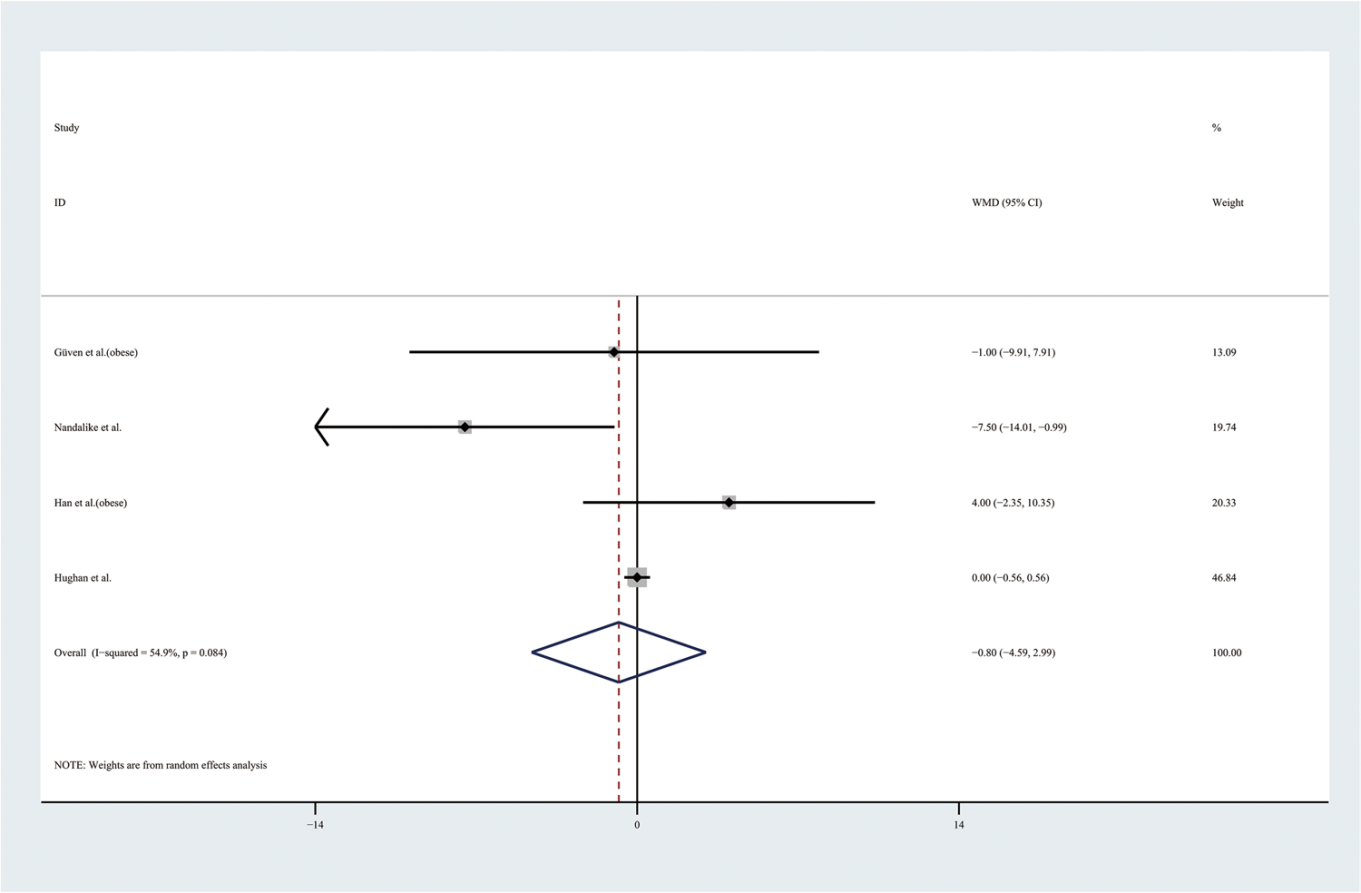
Subgroup analysis of HDL level in obese adolescents with and without PCOS. HDL, high-density lipoprotein; WMD, weighted mean difference; 95% CI, 95% confidence interval. The studies with obese weight were taken to analyse

Therefore, the results of the meta-analysis of 9 studies with a random effect model found no significant differences in TGs levels or HDL levels (Figs. 9 and 10). Considering the results indicating significant statistical heterogeneity, subgroup analyses with the random effect model were only performed in 4 studies of obese adolescents because the number of studies with normal weight adolescents was small (Figs. 11 and 12). The results showed that the level of TGs was higher in obese adolescents with PCOS than in controls (WMD 27.84, 95% CI [10.16, 45.51]) with low statistical heterogeneity (*I*^2^ = 46.1%, *P* = 0.135); however, the level of HDL was not significantly different in the obese groups.

#### Fasting Blood Glucose Level in Adolescents

Ten of the included studies compared the level of FBG between adolescents with PCOS and controls. Two studies found that adolescents with PCOS had higher FBG levels than controls [19, 24], and the remaining 8 studies found no significant differences between groups [16, 18, 20–23, 25, 26]. One study [22] was excluded from the meta-analysis due to a lack of standard deviation data.

**Fig. 13:**
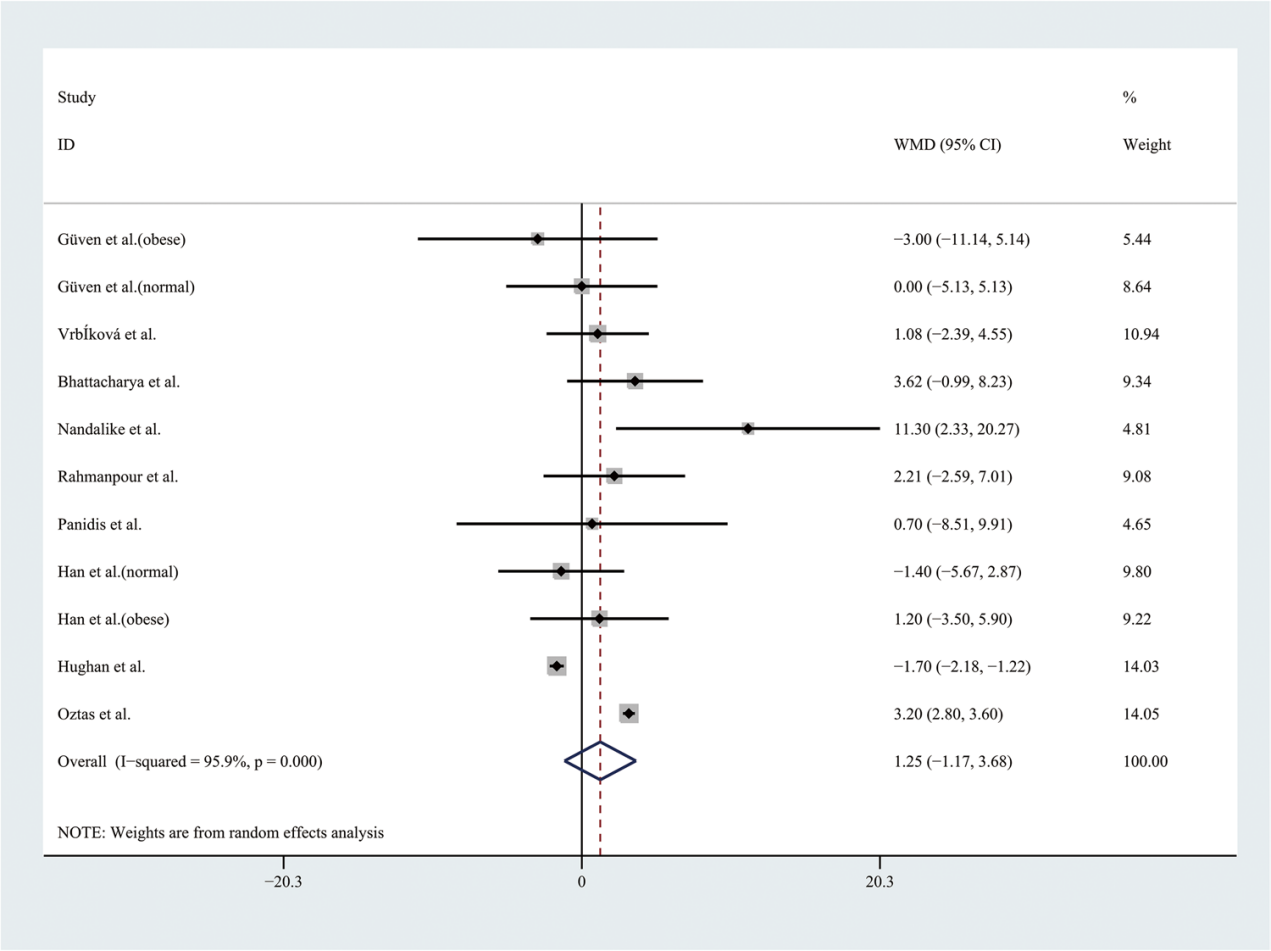
Meta-analysis of FBG level in adolescents with and without PCOS. FBG, fasting blood glucose; WMD, weighted mean difference; 95% CI, 95% confidence interval. Two studies recording the FBG for adolescents of different weight were analysed separately

**Fig. 14:**
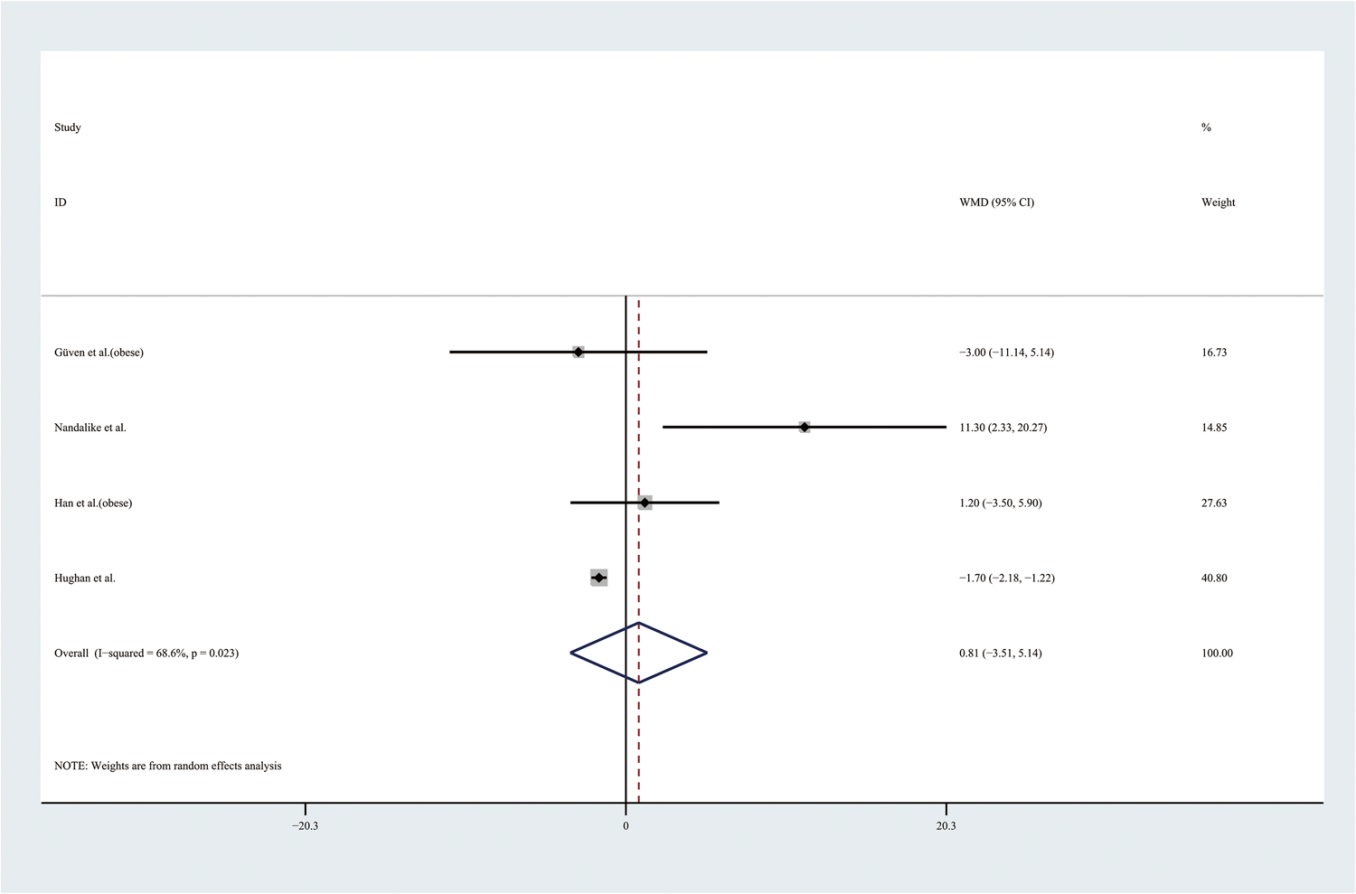
Subgroup analysis of FBG level in obese adolescents with and without PCOS. FBG, fasting blood glucose; WMD, weighted mean difference; 95% CI, 95% confidence interval. The studies with obese weight were taken to analyse

Therefore, the results of the meta-analysis of 9 studies with a random effect model did not find a significant difference in FBG levels (Fig. 13). Subgroup analyses with the random effect model were also performed with 4 studies of obese adolescents (Fig. 14). However, the level of FBG still exhibited no significant differences between obese groups.

### Sensitivity Analysis and Publication Bias

Significant heterogeneity was found in the meta-analysis of all the components of MetS. Therefore, sensitivity analysis was conducted to evaluate the stability of the results by calculating the remaining pooled effect after excluding one study (Figs. S1–S6, shown in supplementary figures). All the results showed that the remaining pooled effect was in the 95% CI of the total pooled effect after excluding one study, which indicated stable results of the meta-analysis of all the components of MetS.

In the meta-analysis of 10 included studies of the prevalence of MetS in adolescents with PCOS, there was no publication bias (Egger’s test = 0.748 and Begg’s test = 1.000). For the components of MetS in adolescents with PCOS, 9 included studies about BP level had no publication bias (SBP, Egger’s test = 0.709 and Begg’s test = 0.350; DBP, Egger’s test = 0.533 and Begg’s test = 0.495), 9 included studies about WC also had no publication bias (Egger’s test = 0.218 and Begg’s test = 0.876), and 9 included studies about lipids’ level and FBG also had no publication bias (TGs, Egger’s test = 0.659 and Begg’s test = 0.119; HDL, Egger’s test = 0.880 and Begg’s test = 0.876; FBG, Egger’s test = 0.954 and Begg’s test = 1.000).

## Discussion

It was reported that women with PCOS were at increased risk for cardiovascular-related adverse outcomes, including high blood pressure, obesity, and metabolic syndrome [29]. Studies have been performed to compare the prevalence of MetS between women with PCOS and healthy controls, and both adults and adolescents with PCOS had a higher risk of MetS [6, 8]. Therefore, we conducted this systematic review and meta-analysis to provide more comprehensive results about the association of PCOS and MetS during adolescence. The results showed that adolescents with PCOS had a more than three-fold higher risk of MetS than controls, and obese adolescents with PCOS also had a higher risk of MetS after matching for BMI, although normal weight adolescents had no significant differences. In addition, higher systolic blood pressure was found in adolescents with PCOS, and diastolic blood pressure was higher in girls with PCOS who had a normal weight, but the levels of triglycerides were higher in obese adolescents with PCOS than in obese adolescents without PCOS, indicating that high blood pressure and dyslipidaemia were likely to be the primary risk factors for MetS in adolescents with PCOS.

On the one hand, the pathogenesis of PCOS was hypothesized to be deriver from functional ovarian hyperandrogenism PCOS [3], and hyperandrogenism could be an independent risk factor of PCOS leading to MetS [30]. On the other hand, insulin resistance and hyperinsulinemia were also commonly found in women with PCOS [31], which could cause vascular muscle wall hypertrophy by interfering with endothelium-dependent vasodilatation mechanisms, leading to hypertension [32]. Even at a young age, adolescents with PCOS manifest significant insulin resistance and impaired glucose tolerance [33] and have increased risk factors for cardiovascular disease compared with controls [26, 34]. Furthermore, insulin resistance might have adverse effects on lipid metabolism [35], including reducing lipid mobilization and attenuating fat oxidation, and lead to metabolic inflexibility in obese adolescents with PCOS [36].

Our findings suggested that PCOS might be an independent risk factor for MetS development in adolescence, and obesity had a significant interaction for this effect. Moreover, the risk of MetS in adolescents with PCOS seemed comparable with that in adults, which indicates the need for effective interventions at an early time. In addition, PCOS might adversely influence blood pressure and lipid metabolism to increase the risk of MetS, implying that periodic detection of blood pressure and biomarkers of lipid metabolism should be considered. Finally, there was no significant publication bias in our analysis. The sensitivity analysis showed clear stability of the results of the meta-analysis.

However, the present review had several limitations. First, subgroup analysis was only based on BMI due to the limited number of included studies, and various subgroup analyses needed to be performed based on ethnicity and the diagnostic criteria of PCOS and MetS in future studies. Second, a higher risk of MetS was mainly found in obese adolescents with PCOS, and lean adolescents had no positive findings. It was previously found that visceral adipose tissue was associated with MetS and might be ignored when accessing BMI, which would not address adiposity distribution in adolescents with normal weight [8].

## Conclusion

In adolescents, PCOS poses a risk of MetS even when controlling for obesity. PCOS might induce hypertension and dyslipidaemia independent of obesity. Therefore, for the adolescents with PCOS, clinicians should not only focus on the management of PCOS but also pay attention to monitoring MetS development. Timely interventions and treatments need to be implemented to improve the long-term prognosis in adolescents with PCOS.

## Supplementary Information

Below is the link to the electronic supplementary material.

Supplementary file1 (PDF 515 KB)

Supplementary file2 (DOCX 38 KB)

## Data Availability

Data transparency.
