# Supplementary material for: The Association Between Polycystic Ovary Syndrome and Metabolic Syndrome in Adolescents: a Systematic Review and Meta-analysis
Source: Reprod Sci. 2022 Feb 2;30(1):28–40. doi: 10.1007/s43032-022-00864-8 (PMC9810687; doi:10.1007/s43032-022-00864-8)
Supplement: Supplementary file 1 — Supplementary file1 (PDF 515 KB) [file 43032_2022_864_MOESM1_ESM.pdf]

Supplementary figures

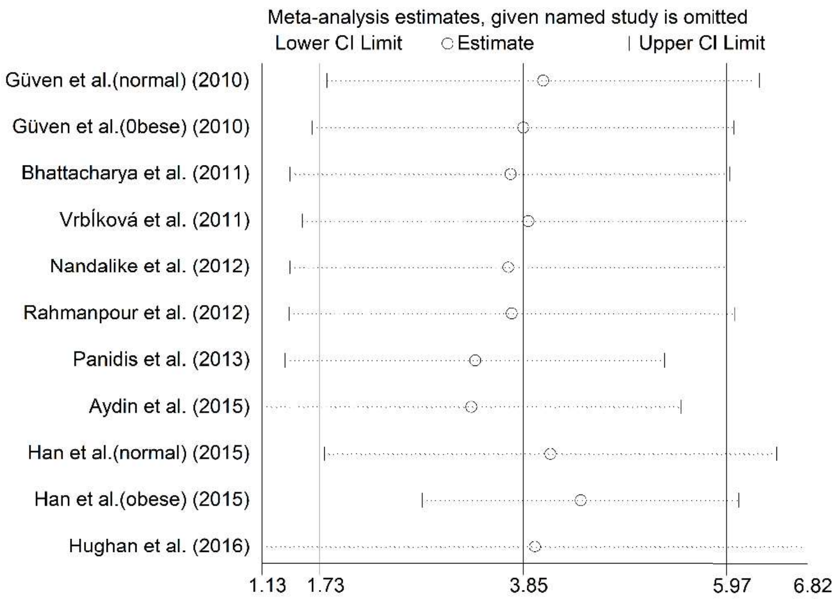

Fig. S1 Sensitivity analysis of SBP level in adolescents with and without PCOS

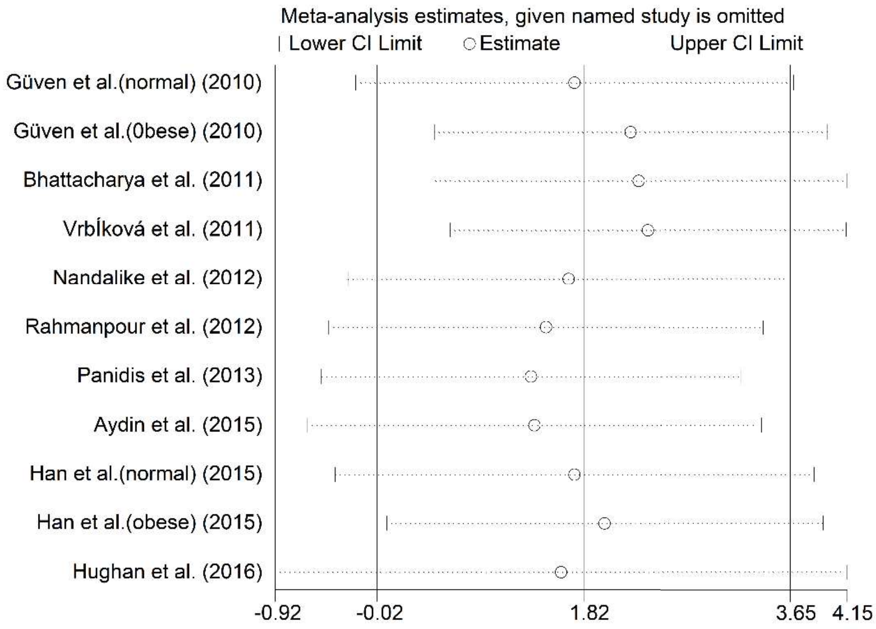

Fig. S2 Sensitivity analysis of DBP level in adolescents with and without PCOS

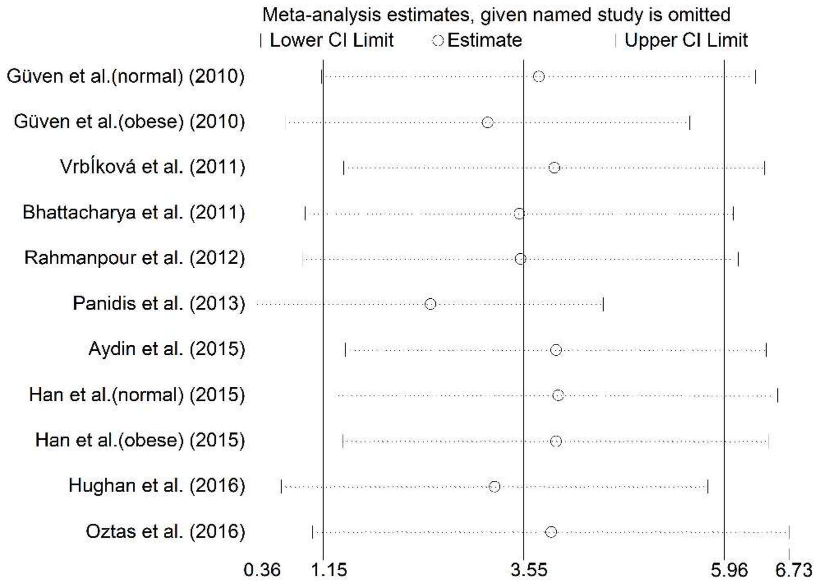

Fig. S3 Sensitivity analysis of WC in adolescents with and without PCOS

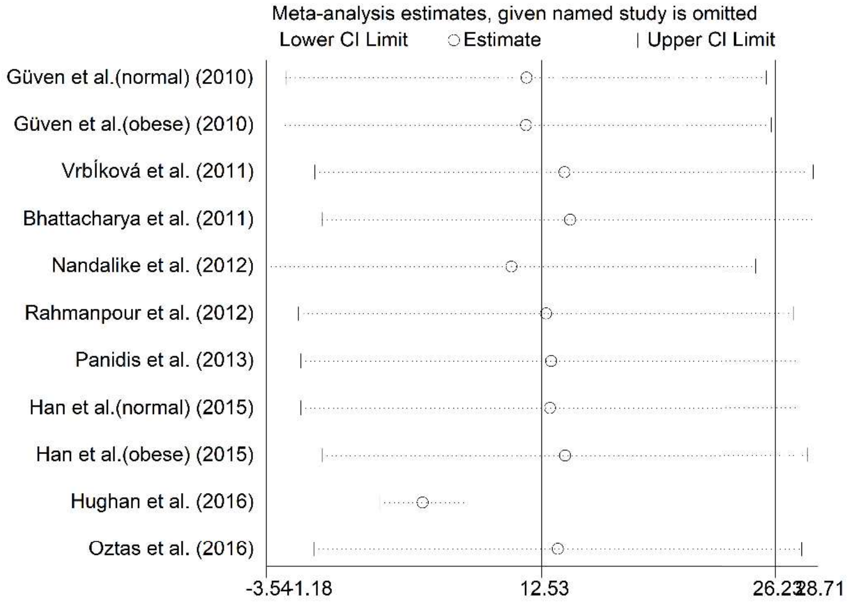

Fig. S4 Sensitivity analysis of TG in adolescents with and without PCOS

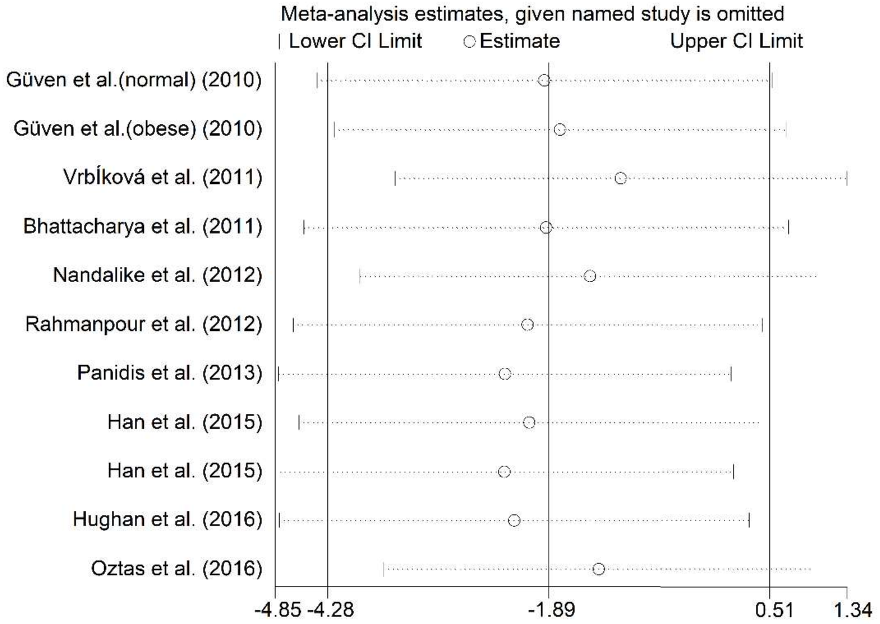

Fig. S5 Sensitivity analysis of HDL in adolescents with and without PCOS

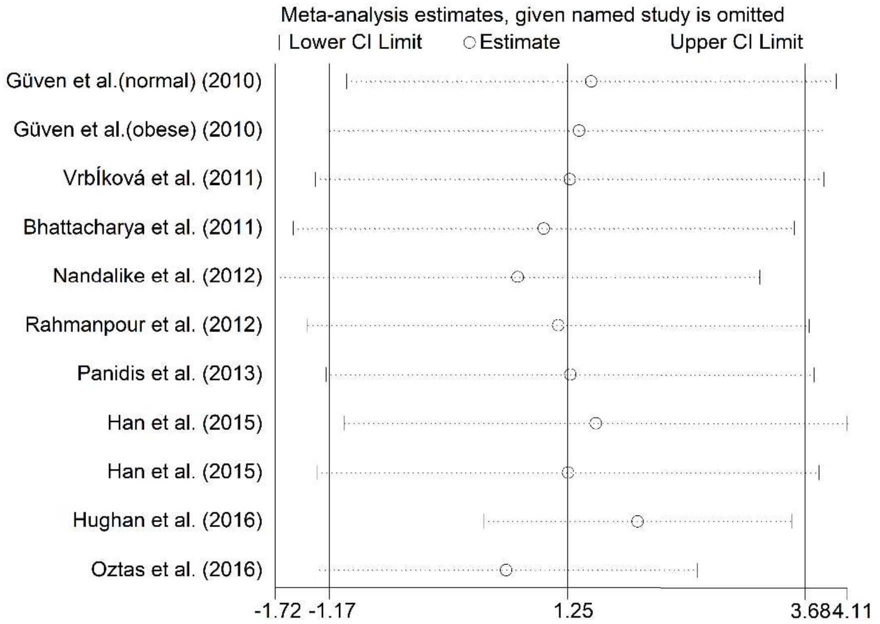

Fig. S6 Sensitivity analysis of FBG in adolescents with and without PCOS
