# Supplementary material for: The Association Between Polycystic Ovary Syndrome and Metabolic Syndrome in Adolescents: a Systematic Review and Meta-analysis
Source: Reprod Sci. 2022 Feb 2;30(1):28–40. doi: 10.1007/s43032-022-00864-8 (PMC9810687; doi:10.1007/s43032-022-00864-8)
Supplement: Supplementary file 2 — Supplementary file2 (DOCX 38 KB) [file 43032_2022_864_MOESM2_ESM.docx]

**Table S1** **Characteristics of included studies in the systematic review.**

| Author  (year) | Country | PCOS | Control | MetS definition | MetS prevalence in PCOS vs controls | PCOS vs control for each component of MetS  (Mean±SD) | P value |
| --- | --- | --- | --- | --- | --- | --- | --- |
| Güven et al. (2010) | Turkey | Diagnostic criteria: Rotterdam  N: 22, Mean age: 15.2 years old  Mean BMI: 26.5 kg/m² | N: 16, Mean age: 15.1 years old  Mean BMI: 24.3kg/m² | / | / | Normal weight: (n: 12 vs 9)  WC: 68±3 cm vs 66.1±5 cm  SBP: 112±9 mmHg vs 111±7 mmHg  DBP: 73±7 mmHg vs 70±7 mmHg  TG: 94±43 mg/dL vs 71±25 mg/dL  HDL: 47±12 mg/dL vs 52±13 mg/dL  FBG: 83±7 mg/dL vs 83±5 mg/dL  Obese: (n:10 vs 7)  WC: 96.4±10 cm vs 84.7±7 cm  SBP: 129±17 mmHg vs 125±12 mmHg  DBP: 78±10 mmHg vs 85±5 mmHg  TG: 130±72 mg/dL vs 99±33 mg/dL  HDL: 40±8 mg/dL vs 41±10 mg/dL  FBG: 81±11 mg/dL vs 84±6 mg/dL | NS  NS  NS  NS  NS  NS  P＜0.05  NS  NS  NS  NS  N |
| Huang et al. (2010) | China | Diagnostic criteria: Rotterdam  N: 128, Mean age: 18 years old  Mean BMI: 20 kg/m² | N: 40, Mean age: 19 years old  Mean BMI: 19.6 kg/m² | IDF（10-16）  IDF（＞16） | 4.69% vs 2.50%  P value: N/A | / |  |
| Bhattacharya et al. (2011) | India | Diagnostic criteria: AES 2006 criteria  N: 51, Mean age: 17.06 years old  Mean BMI: 26.37 kg/m² | N: 45, Mean age: 16.69 years old  Mean BMI: 25.38 kg/m² | 2009 ‘joint interim criteria’ | 60.78% vs 26.67%  P value: 0.002 | WC: 81.54±9.9 cm vs 77.38±10.2 cm  SBP: 124.04±16.4 mmHg vs 118.62±12.4 mmHg  DBP: 78.16±8.7 mmHg vs 80.22±7.6 mmHg  TG: 136.06±61.7 mg/dL vs 138.64±20.2 mg/dL  HDL: 39.1±8.8 mg/dL vs 40.8±3.9 mg/dL  FBG: 88.04±11.4 mg/dL vs 84.42±11.6 mg/dL | P＜0.05  NS  NS  NS  NS  NS |
| Hart et al.  (2011) | Australia | Diagnostic criteria: Rotterdam  N: 61, Mean age: 15.1 years old  Mean BMI: 24.2 kg/m² | N: 143, Mean age: 15.1 years old  Mean BMI: 22.1 kg/m² | IDF（10-16） | 4.92% vs 4.20%  P value: 0.999 | / |  |
| VrbÍková et al. (2011) | Czech Republic | Diagnostic criteria: ESHRE/ASRM  N: 43, Mean age: 16.84 years old  Mean BMI: 23.64 kg/m² | N: 48, Mean age: 17.5 years old  Mean BMI: 23.37 kg/m² | IDF | 11.63% vs 2.08%  P value: N/A | WC: 75.79±15.46 cm vs 76.77±10.75 cm  SBP: 116.66±13.24 mmHg vs 113.21±10.72 mmHg  DBP: 72.37±9.14 mmHg vs 75.74±9.43 mmHg  TG: 86.83±37.21 mg/dL vs 85.94±41.64 mg/dL  HDL: 52±14.36 mg/dL vs 63.63±16.68 mg/dL  FBG: 86.22±9 mg/dL vs 85.14±7.74 mg/dL | NS  NS  NS  NS  P＜0.05  NS |
| Nandalike et al. (2012) | USA | Diagnostic criteria: Rotterdam  N: 28, Mean age: 16.8 years old  Mean BMI: 44.8 kg/m² | N: 28, Mean age: 17.1 years old  Mean BMI: 40.2 kg/m² | Weiss criteria | 35.71% vs 14.29%  P value: 0.1 | WC: /  SBP: 122.9±13.6 mmHg VS 117.1±9.6 mmHg  DBP: 69.8±11.2 mmHg VS 66.4±8.4 mmHg  TG: 125.5±73.1 mg/dL VS 91.4±38.9 mg/dL  HDL: 43±10.9 mg/dL vs 50.5±13.8 mg/dL  FBG: 96.6±20.1 mg/dL vs 85.3±13.5 mg/dL | /  P＜0.05  NS  NS  P＜0.05  P＜0.05 |
| Rahmanpour et al. (2012) | Iran | Diagnostic criteria: NIH  N: 30, Mean age: 17.73 years old  Mean BMI: 23.4kg/m² | N: 71, Mean age: 17.69 years old  Mean BMI: 21.06kg/m² | IDF（＞16） | 33.33% vs 11.27%  P value: 0.038 | WC: 74.3±7.26 cm vs 70.31±7.91 cm  SBP: 108.17±12.06 mmHg vs 103.1±12.22 mmHg  DBP: 76.67±11.24 mmHg vs 70.83±11.43 mmHg  TG: 108.23±59.88 mg/dL vs 98.55±40.87 mg/dL  HDL: 35.53±12.53 mg/dL vs 35.38±4.32 mg/dL  FBG: 96.6±12.32 mg/dL vs 94.39±8.21 mg/dL | P＜0.05  NS  P＜0.05  P＜0.05  NS  NS |
| Panidis et al. (2013) | Greece | Diagnostic criteria: Rotterdam  N: 332, Mean age: 18.1 years old  Mean BMI: 27.8kg/m² | N: 10, Mean age: 18.3 years old  Mean BMI: 21.3kg/m² | IDF | 30.12% vs 0.00%  P value: NS | WC: 85.3±15.2 cm vs 70.6±5.8 cm  SBP: 106.9±15.2 mmHg vs 95±11 mmHg  DBP: 68.6±12 mmHg vs 61±7.4 mmHg  TG: 87.9±40.4 mg/dL vs 80.3±26.9 mg/dL  HDL: 38.9±13.1 mg/dL vs 33±12.7 mg/dL  FBG: 97.4±12.4 mg/dL vs 96.7±14.7 mg/dL | P＜0.01  P＜0.05  P＜0.05  NS  NS  NS |
| Aydin et al. (2015) | Turkey | Diagnostic criteria: ESHRE/ASRM  N: 63, Mean age: 15.72 years old  Mean BMI: 20.23 kg/m² | N: 159, Mean age: 16.37 years old  Mean BMI: 21.03 kg/m² | modified Cook criteria | 7.94% vs 0.63%  P value: 0.02 | WC: 77.49±21.36 cm vs 78.73±10.22 cm  SBP: 112.14±12.64 mmHg vs 104.49±13.93 mmHg  DBP: 74.12±9.09 mmHg vs 69.01±11.29 mmHg  TG: 91.22 mg/dL vs 78.91 mg/dL  HDL: 61.09 mg/dL vs 57.98 mg/dL  6.FBG: 84.44 mg/dL vs 85.91 mg/dL | NS  P＜0.05  P＜0.05  P＜0.05  NS  NS |
| Han et al.  (2015) | Korea | Diagnostic criteria: NIH  N: 49, Mean age: 17.1 years old  Mean BMI: 22.9kg/m² | N: 40, Mean age: 16.9 years old  Mean BMI: 22.1kg/m² | NCEP ATP III criteria | 4.08% vs 2.50%  P value: N/A | Normal weight: (n: 25 vs 25)  WC: 70.8±3.9 cm vs 70.8±6.5 cm  SBP: 108.4±8.8 mmHg vs 106.8±6.7 mmHg  DBP: 69.8±3.2 mmHg vs 67.6±6.1 mmHg  TG: 87.9±33.5 mg/dL vs 80±34.2 mg/dL  HDL: 51.3±10 mg/dL vs 50.6±12.9 mg/dL  FBG: 86.9±8.1mg/dL vs 88.3±7.3 mg/dL  Obese: (n:24 vs 15)  WC: 81.8±5.5 cm vs 82.3±7.6 cm  SBP: 109.5±10.1 mmHg vs 115.3±8.1 mmHg  DBP: 81.8±5.5 mmHg vs 82.3±8.6 mmHg  TG: 104.7±51.9 mg/dL vs 111.9±57.7 mg/dL  HDL: 47.1±10.8 mg/dL vs 43.1±9.2 mg/dL  FBG: 86.4±7.1mg/dL vs 85.2±7.4 mg/dL | NS  NS  NS  NS  NS  NS  NS  NS  P＜0.05  NS  NS  NS |
| Oztas et al.  (2016) | Turkey | Diagnostic criteria: ESHRE/ASRM  N: 89, Mean age: 18.4 years old  Mean BMI: 24.6 kg/m² | N: 83, Mean age: 18.8 years old  Mean BMI: 21.4 kg/m² | IDF（＞16） | 10.11% vs 6.02%  P value: NS | WC: 78.5±1.45 cm vs 77.3±1.45 cm  SBP: /  DBP: /  TG: 95.2±8.7 mg/dL vs 89.8±9 mg/dL  HDL: 62.3±1.75 mg/dL vs 66.7±1.85 mg/dL  FBG: 88.7±1.35 mg/dL vs 85.5±1.35 mg/dL | NS  /  /  NS  P＜0.05  P＜0.05 |
| Hughan et al. (2016) | USA | Diagnostic criteria: ESHRE/ASRM  N: 91, Mean age: 15.8 years old  Mean BMI: 37.9kg/m² | N: 30, Mean age: 14.6 years old  Mean BMI: 35.3kg/m² | / | / | WC: 107.6±2 cm vs 101.4±3.6 cm  SBP: 123.3±1.4 mmHg vs 120±2.6 mmHg  DBP: 64.9±0.8 mmHg vs 62.3±1.3 mmHg  TG: 119.7±6.3 mg/dL vs 83.8±6.7 mg/dL  HDL: 42.9±1.2 mg/dL vs 42.9±1.4 mg/dL  FBG: 87.2±1 mg/dL vs 88.9±1.2 mg/dL | P＜0.05  P＜0.05  NS  P＜0.05  P＜0.05  NS |

Note: PCOS: polycystic ovary syndrome; MetS: metabolic syndrome; NIH: National Institutes of Health; ESHRE/ASRM: European Society for Human Reproduction and Embryology and American Society for Reproductive Medicine; AES: Androgen Excess society; IDF: International Diabetes Federation; NCEP ATP III Criteria: The National Cholesterol Education Program Adult Treatment Panel III criteria; WC: waist circumference; SBP: systolic blood pressure; DBP: diastolic blood pressure; TG: triglycerides; HDL: high-density lipoprotein; FBG: fasting blood glucose; SD: standard difference; NS: no significance. In the twelve studies, ten studies compared the prevalence of MetS in adolescents with PCOS and controls, the other 2 studies only included all the components of MetS in in adolescents with PCOS and controls. Some studies matched BMI by dividing weight into normal weight and obese weight for research.

**Table S2 Quality assessment of included studies using the Newcastle- Ottawa Quality Assessment Scale**

| Author (year) | Study design | Selection (max 4 stars) | Comparability (max 2 stars) | Exposure (max 3 stars) | Overall quality |
| --- | --- | --- | --- | --- | --- |
| Güven et al. (2010) | Case-control study | ** | ** | ** | Fair |
| Huang et al. (2010) | Case-control study | **** | ** | ** | Good |
| Bhattacharya et al. (2011) | Case-control study | **** | * | ** | Good |
| Hart et al. (2011) | Prospective cohort study | **** | * | *** | Good |
| VrbÍková et al. (2011) | Case-control study | ** | * | ** | Fair |
| Nandalike et al. (2012) | Case-control study | ** | * | ** | Fair |
| Rahmanpour et al. (2012) | Case-control study | ** | ** | ** | Fair |
| Panidis et al. (2013) | Case-control study | ** | * | ** | Fair |
| Aydin et al. (2015) | Case-control study | ** | ** | ** | Fair |
| Han et al. (2015) | Case-control study | *** | ** | ** | Good |
| Oztas et al. (2016) | Case-control study | ** | * | ** | Fair |
| Hughan et al. (2016) | Case-control study | ** | ** | ** | Fair |
